# Correction: Ionic supramolecular polymerization of water-soluble porphyrins: balancing ionic attraction and steric repulsion to govern stacking

**DOI:** 10.1039/d4ra90010c

**Published:** 2024-02-05

**Authors:** Chisako Kanzaki, Hiroshi Yoneda, Shota Nomura, Takato Maeda, Munenori Numata

**Affiliations:** a Department of Biomolecular Chemistry, Graduate School of Life and Environmental Sciences, Kyoto Prefectural University Shimogamo, Sakyo-ku Kyoto 606-8522 Japan numata@kpu.ac.jp +81-75-703-5132

## Abstract

Correction for ‘Ionic supramolecular polymerization of water-soluble porphyrins: balancing ionic attraction and steric repulsion to govern stacking’ by Chisako Kanzaki *et al.*, *RSC Adv.*, 2022, **12**, 30670–30681, https://doi.org/10.1039/D2RA05542B.

The authors regret that an incorrect version of Fig. 1 was included in the original article. The correct version of Fig. 1 is presented below.

**Fig. 1:**
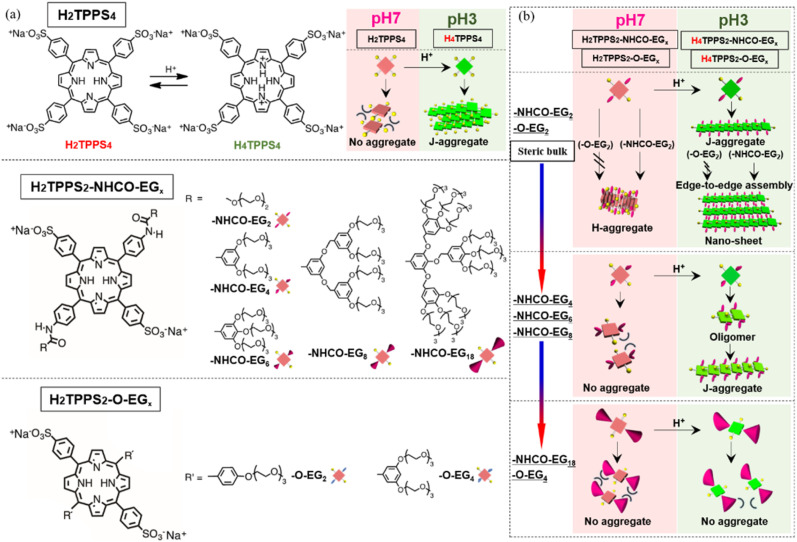
(a) Molecular structures of H_2_TPPS_4_, H_2_TPPS_2_-NHCO-EG_*x*_ (*x* = 2, 4, 6, 8, 18), and H_2_TPPS_2_-O-EG_*x*_ (*x* = 2, 4); the DFT-calculated structures of the H_2_TPPS_2_-NHCO-EG_*x*_ (*x* = 2, 4, 6, 8, 18) and H_2_TPPS_2_-O-EG_*x*_ (*x* = 2, 4) derivatives are provided in the ESI (Fig. S18). (b) Schematic representation of EG unit dependent supramolecular polymerization toward H- and J-aggregates. Synthetic procedures and spectral data for these porphyrins are available in the ESI.

The Royal Society of Chemistry apologises for these errors and any consequent inconvenience to authors and readers.

## Supplementary Material

